# Selective Inhibitors of Nuclear Export (SINE) compounds block proliferation and migration of triple negative breast cancer cells by restoring expression of ARRDC3

**DOI:** 10.18632/oncotarget.17987

**Published:** 2017-05-18

**Authors:** Young Hwa Soung, Trinayan Kashyap, Thalia Nguyen, Garima Yadav, Hua Chang, Yosef Landesman, Jun Chung

**Affiliations:** ^1^ Department of Pathology, Stony Brook Medicine, Stony Brook, NY 11794, USA; ^2^ Karyopharm Therapeutics, Inc. Newton, MA 02459, USA; ^3^ University of Oklahoma Health Science Center, Oklahoma City, OK 73104, USA

**Keywords:** arrestin related domain containing 3 (ARRDC3), Selective Inhibitors of Nuclear Export (SINE) compounds, selinexor, exportin-1 (XPO-1), triple negative breast cancer (TNBC)

## Abstract

Arrestin-related domain-containing protein-3 (ARRDC3) is one of 6 mammalian arrestins, which suppresses metastasis by inducing degradation of phosphorylated β2-adrenergic receptor (β2 AR) and integrin β4 (ITG β4). Our previous studies demonstrated that expression of ARRDC3 is epigentically silenced in Triple Negative Breast Cancer (TNBC) cells, and the forced expression of ARRDC3 significantly reduced the invasive potential of TNBC cells. In the current study, we found that Selective Inhibitors of Nuclear Export (SINE) compounds (KPT-185 and selinexor (KPT-330)) restore ARRDC3 expression in TNBC cell lines (MDA-MB-231 and MDA-MB-468) at both the mRNA and protein level in a dose and time course dependent manner. SINE compounds inhibit the proliferation, pro-invasive migration and anchorage independent growth of the TNBC cells by restoring ARRDC3 expression. We found that ARRDC3 expression is lower in TNBC cell lines than those of luminal breast cancer cell lines, and inversely correlated with IC_50s_ of selinexor. Analysis of tissue microarray confirmed that ARRDC3 expression in patient samples is significantly lower in the majority of TNBC tumors relative to normal tissue. *In vivo*, selinexor inhibited the tumor growth of MDA-MB-231 xenografts by nearly 100% compared with vehicle treated animals. Furthermore, immunohistochemical analysis of TNBC tumors from selinexor treated mice revealed increased ARRDC3 expression versus vehicle treated animals. Our results suggest that restoration of ARRDC3 expression is an important antineoplastic mechanism of SINE compounds in TNBC, and therefore selinexor could be an effective treatment option for breast tumors with down-regulated ARRDC3.

## INTRODUCTION

The incidence of breast cancer has increased over the past 10 years and the survival rate of patients diagnosed with metastatic breast cancer have been dramatically dropping despite the significant advances in treatment and detection of breast cancer [1, 2]. Approximately 15–20% of metastatic breast carcinoma is defined as triple negative breast cancer (TNBC) [1, 2]. Among the four district sub-types (luminal A, luminal B, HER2-positive and TNBC) classified by gene expression profiles, TNBC is the most aggressive type with the worst clinical outcome [3–5]. Currently, there is no approved targeted therapy for either early or late stage TNBC patients, as a majority of TNBC lacks therapeutically targetable hormone receptors (estrogen and progesterone) and HER2 [6–8]. Some TNBC-targeted therapeutics including cetuximab (anti-EGFR monoclonal antibody), imatinib (c-KIT tyrosine kinase inhibitor), iniparib (PARP inhibitor) and cisplatin are currently undergoing preclinical/clinical investigation [9–13], but clinical trials of these agents have failed to demonstrate the therapeutic efficacy. For this reason, discovering effective and druggable molecular targets for TNBC is an urgent issue.

Our previous studies demonstrated that epigenetic silencing of ARRDC3 is linked to the aggressive nature of TNBC cells [14], suggesting that ARRDC3 could be a novel therapeutic target for TNBC. ARRDC3 is a negative regulator of β2-adrenergic receptor (β2 AR) and integrin β4 (ITG β4), which mediates the ubiquitination and subsequent degradation of the phosphorylated forms of these receptors [15–18]. A negative regulation of β2 AR and ITG β4, whose roles in breast cancer progression are established, by ARRDC3 indicates its role as a potential metastatic suppressor [14, 16]. Therefore, the identification of small molecule based drugs that restore the expression of ARRDC3 merit consideration as a therapeutic option for TNBC and SINE compounds are such candidates.

SINE compounds, are small molecule inhibitors of Exportin 1 (XPO1, chromosome region maintenance 1, CRM1). Expression of XPO1 is up-regulated in several types of cancer and its overexpression is linked to cancer cell survival, proliferation and drug resistance [19–21]. SINE compounds covalently bind to Cysteine 528 residue in the central conserved cargo binding region of XPO1 and inhibit its function [22]. This leads to the forced nuclear retention of tumor suppressor proteins such as p53 [23], IkB [24], and FOXO [25], and subsequently induces their tumor suppressive actions leading to cell cycle arrest and cell death [24–27]. This activity is ablated by mutating Cysteine 528, showing the high drug-target selectivity [22]. More recent evidence demonstrated the anti-cancer effects of SINE compounds *in vitro* and *in vivo* for hematological and solid tumors [28–33] including phase I clinical studies [34–37]. However, SINE compounds as a therapeutic option for breast cancer has not been extensively studied.

In this study, we investigated the anti-cancer effects of SINE compounds on TNBC models *in vitro* and *in vivo* and evaluated the role of ARRDC3 in mediating anti-cancer effects of SINE compounds. The data presented here suggests that SINE compounds can be a promising therapeutic option for the sub-type of TNBC with downregulated ARRDC3 expression.

## RESULTS

### SINE compounds restore ARRDC3 expression in TNBC cell lines

**Figure 1:**
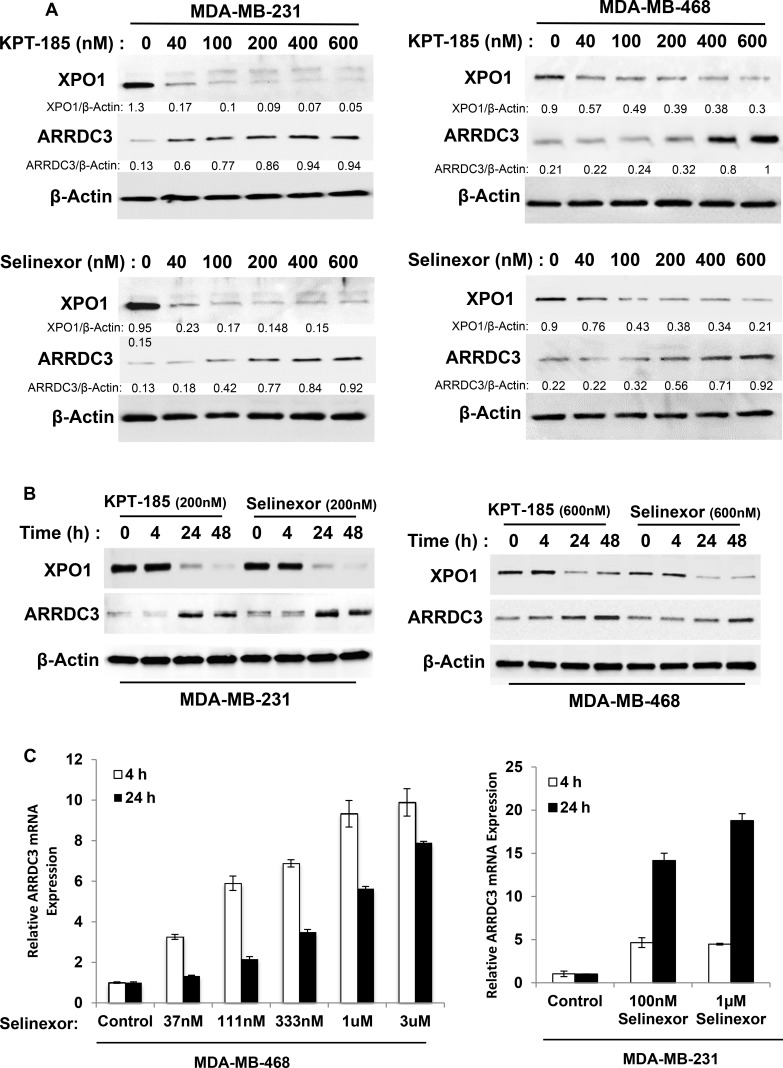
SINE compounds restore ARRDC3 expression in TNBC cell lines (**A**) MDA-MB-231 and MDA-MB-468 cells were treated with or without various concentrations of KPT-185 and KPT-330 (selinexor) for 24 h prior to lysis by RIPA buffer. Whole cell lysates were prepared and analyzed to measure the levels of ARRDC3, XPO1 and β-actin by western blotting. Densitometric analysis was performed to measure the relative intensity of the bands after normalizing with β-actin. The fold changes were indicated below each lane. (**B**) MAD-MB-231 cells and MDA-MB-468 cells were treated with SINE compounds (200 nM and 600 nM respectively) at the indicated times. Protein levels of ARRDC3, XPO1 and β-actin were determined by western blot analysis. (**C**) Cells were treated with selinexor at the indicated concentrations for 4 h and 24 h and then RNAs were prepared as described in the Material and Methods. ARRDC3 mRNA levels were determined by qRT-PCR. Blot images and graphs are representative data of three independent experiments.

Our previous studies demonstrated that ARRDC3 expression is epigenetically silenced in invasive TNBC cells [14] and the forced expression of ARRDC3 inhibited the cell motility and proliferation of TNBC [14, 17]. Therefore, we hypothesized that small molecule compounds restoring ARRDC3 expression in TNBC cells could potentially be a novel therapeutic option for TNBC. We tested KPT-185 and selinexor (KPT-330), two SINE compounds that based on gene chip analysis, were found to induce ARRDC3 transcript levels in the fibrosarcoma cell line HT1080. We tested two TNBC cell lines (MDA-MB-231 and MDA-MB-468) that have low basal levels of ARRDC3 expression. We found that treatment of SINE compounds for 24 hours significantly increased the ARRDC3 protein levels in both TNBC cell lines in a dose-dependent manner (Figure 1A). Time course studies showed that at least 4–24 hours were required for SINE compounds to restore the ARRDC3 protein levels in the TNBC cell lines (Figure 1B). Quantitative PCR analysis confirmed these findings by showing that treatment of SINE compounds (as early as 4 hours) induced ARRDC3 mRNA levels in both MDA-MB-231 and MDA-MB-468 cells (Figure 1C). Induction of ARRDC3 expression by SINE compounds is not due to apoptosis or cell death (Supplementary Figure 1), and is directly related to inhibition of XPO1 function as knocking down XPO1 expression by siRNA results with upregulation of ARRDC3 expression (Supplementary Figure 2).

### SINE compounds inhibit the tumorigenic and metastatic potential of TNBC in an ARRDC3 dependent manner

**Figure 2:**
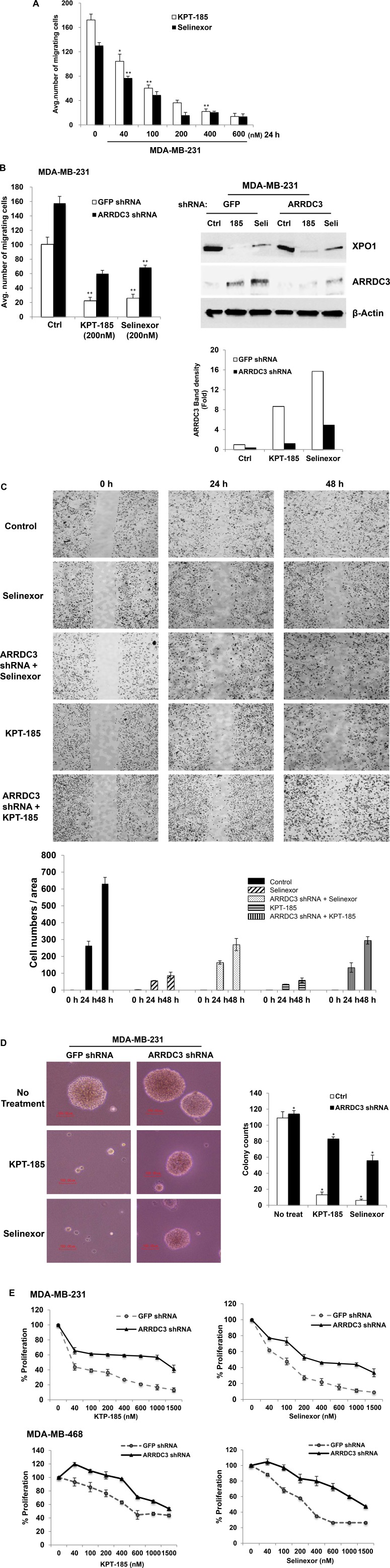
SINE compounds inhibit the tumorigenic and metastatic potential of TNBC in an ARRDC3 dependent manner (**A**) MDA-MB-231 cells were treated with various concentrations of KPT-185 and selinexor. The ability of cells to migrate toward 100 nM LPA was measured using a transwell cell motility assay after 24 h treatment. Migration was quantified by counting the cells that migrated to the lower surface of the membrane per square milliliter using bright-field optics. (**B**) MDA-MB-231 cells were stably infected with either GFP (as control) or ARRDC3 shRNA. These cells were treated with 200 nM of KPT-185 and selinexor for 24 h and subjected to transwell migration (Left panel). Equal amounts of proteins from each sample were used for western blot analysis with antibodies against ARRDC3, XPO1 and β-actin (Right panel). The band intensities were measured by software ImageJ. (**C**) The MDA-MB-231 cells expressing shRNA against GFP and ARRDC3 were loaded into the chambers (Ibidi's culture-insert in μ-dish) and allowed to adhere overnight. The chambers were removed and then SINE compounds were added to dish. Snapshots at specific time points were used as representative image. Cells migrated into the wound area were counted by Fiji. Representative images were carried out in triplicate. (**D**) MDA-MB-231 cells expressing GFP or ARRDC3 shRNA were cultured in soft agar containing growth medium with KPT-185 and selinexor for two weeks as described in materials and methods. Left panel shows images of colony conformation, which is captured at 10× magnification. Right graph shows quantification of colony numbers. Scale bar: 100 μm. (**E**) MDA-MB-231 and MDA-MB-468 cells expressing GFP or ARRDC3 shRNA were treated for 72 h and 48 h respectively with different concentrations of KPT-185 and selinexor. Proliferation of these cells was measured by MTT assay. Column, mean from three independent experiments; bars, SD. *P < 0.01; **P < 0.001 compared with the control group.

Once we concluded that SINE compounds induced ARRDC3 protein expression, we aimed to determine if SINE compounds inhibit the tumorigenic and metastatic potential of TNBC. Therefore, we started by assessing the inhibitory effects of SINE compounds on TNBC cell motility. We used the trans-well assay and measured cell motility towards the chemo-attractant (lysophosphatidic acid; LPA). LPA. Both KPT-185 and selinexor effectively inhibited MDA-MB-231 cell motility in a dose-dependent manner (Figure 2A). To confirm that SINE compounds-induced-inhibition of cell motility is truly the result of the restoration of ARRDC3 expression, we knocked down ARRDC3 in these cells. As shown in Figure 2B, knockdown of ARRDC3 expression by shRNA in MDA-MB-231 cells prevented SINE compounds mediated restoration of ARRDC3 expression and significantly reduced the inhibitory effects of SINE compounds on TNBC cell motility towards LPA compared to that of the control cells (GFP shRNA cells). We then repeated the assay and demonstrated the anti-migration effects of SINE compounds in TNBC cells by using a different cell motility model, namely a wound healing assay (Figure 2C). As expected, in this system the inhibition of wound healing by SINE compounds in MDA-MB-231 cells was not as effective when restoration of ARRDC3 expression was prevented by shRNA (Figure 2C).

Next, we measured the effects of forced ARRDC3 expression on the anchorage-independent growth of TNBC cells by performing the colony formation assay of MDA-MB-231 cells on soft agar with or without treatment of SINE compounds. Treatment with each of the SINE compounds effectively blocked the colony formation of MDA-MB-231 cells in soft agar in comparison with that of control cells (Figure 2D). ARRDC3 shRNA expression effectively blocked the inhibitory effects of SINE compounds on colony formation whereas it had no effect on colony formation of MDA-MB-231 cells without treatment of SINE compounds (Figure 2D). These results indicate that SINE compounds also require ARRDC3 expression to inhibit anchorage independent growth of MDA-MB-231 cells.

Finally, we tested the effects of SINE compounds on the proliferation of MDA-MB-231 and MDA-MB-468 cell lines and found that both SINE compounds promoted effective cytostatic effects of both cell lines (Figure 2E). Knockdown of ARRDC3 expression by shRNA in both cell lines once again reduced the anti-proliferative effects of SINE compounds (Figure 2E). Inhibition of TNBC cell motility, anchorage independent growth, and proliferation by SINE compounds is not due to apoptosis or cell death (Supplementary Figure 1). We concluded that ARRDC3 expression is central for mediating the anti-cancer effects of SINE compounds.

### TNBC subtype breast cancer cells are more sensitive to the cytotoxic effects of selinexor than luminal subtype breast cancer cells and their sensitivity is inversely correlated with ARRDC3 basal levels of expression

**Figure 3:**
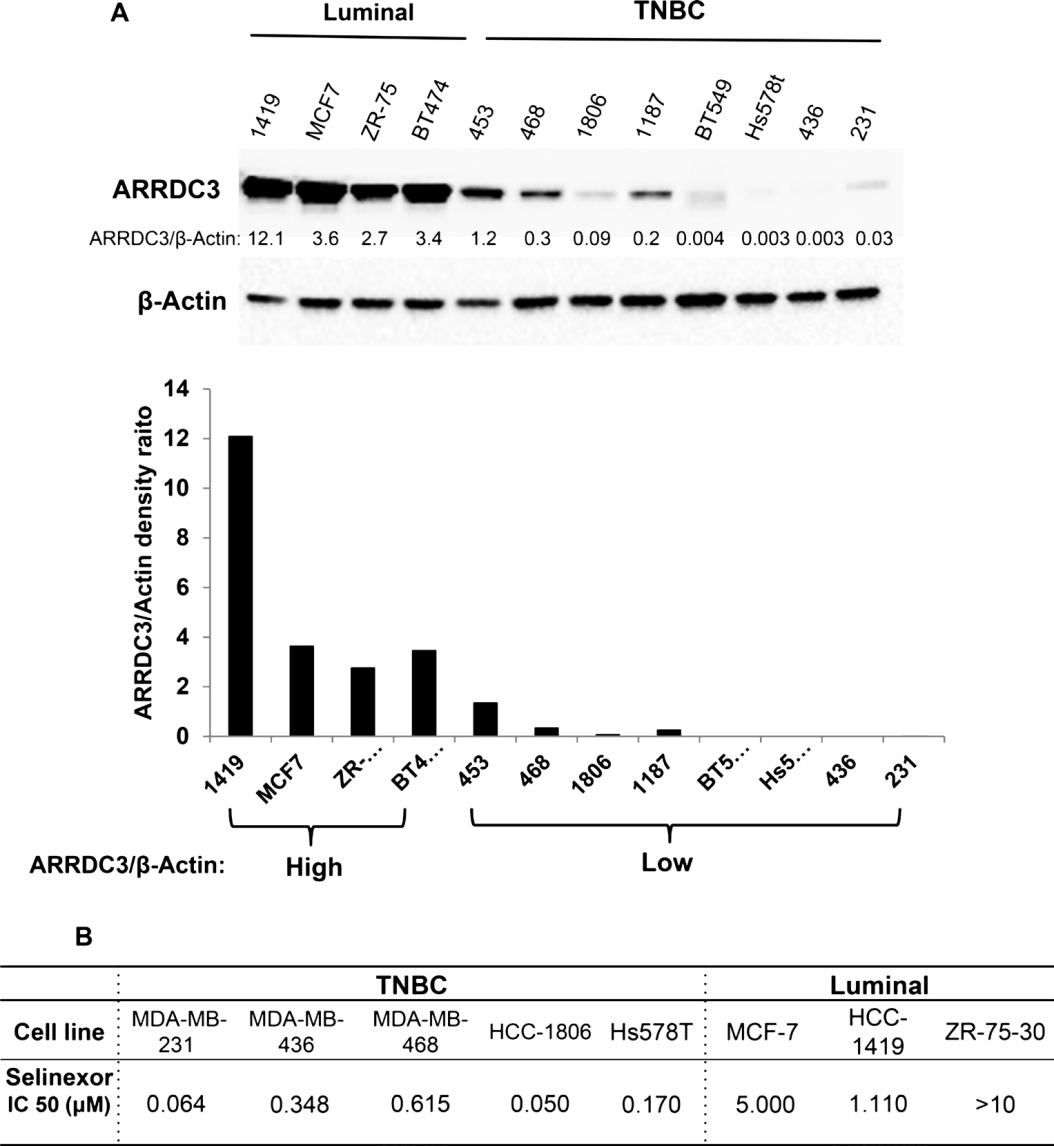
TNBC subtype breast cancer cells are more sensitive to the cytotoxic effects of selinexor than luminal subtype breast cancer cells and their sensitivity is inversely correlated with ARRDC3 basal levels of expression (**A**) Whole cell lysates were prepared from 4 luminal (HCC-1419, MCF-7, ZR-75-30 and BT474) and 8 TNBC cell lines (MDA-MB-453, MDA-MB-468, HCC-1806, HCC-1187, BT549, Hs578T, MDA-MB-436 and MDA-MB-231). Equal amounts of extracts from each sample were used for western blot analysis by using anti-ARRDC3 antibody. β-actin was used as loading control. Densitometric analysis was performed to measure the relative intensity of the bands after normalizing with β-actin. (**B**) Luminal and TNBC cell lines were seeded in 96-well plates and then treated with various concentrations of KPT-185 and selinexor for 72 h. Cell viability was measured by MTT assay. IC_50_ values were determined by using GraphPad Prism 6.

The ability of SINE compounds to induce cytotoxicity in TNBC cells through the induction of ARRDC3 expression suggest that in these sensitive tumor cells, the ARRDC3 mediated pathways are fully active downstream of this tumor suppressor protein. Therefore, we hypothesized that the lowest levels of ARRDC3 may indicate higher SINE compounds sensitivity. To test this hypothesis, we measured the steady state protein levels of ARRDC3 in 12 breast cancer cell lines with different subtypes (4 luminal and 8 TNBC subtypes) (Figure 3A). Based on the densitometric analysis of ARRDC3 and beta-actin bands, we concluded that the protein levels of ARRDC3 are largely divided into high and low groups (Figure 3A). A majority of TNBC cell lines belong to ARRDC3 low expression group (0.003–1.2 scale) whereas luminal subtype of breast cancer cell lines belong to ARRDC3 high expression group (3.4–12.1). We then measured and compared the IC_50s_ of selinexor in the TNBC vs. luminal breast cancer cell lines. As shown in Figure 3B, we found that IC_50s_ of selinexor were significantly lower in TNBC cell lines (0.05–0.615 μM) compared to those of luminal breast cancer cell lines (1.1- over 10 μM). The outcome further supports our hypothesis that selinexor works more efficiently in breast cancers with down-regulated ARRDC3 as restoration of ARRDC3 expression is an important therapeutic mechanism of this drug.

### ARRDC3 expression is low in many TNBC tumors and inversely correlates with the levels of XPO1 and integrin β4

**Figure 4:**
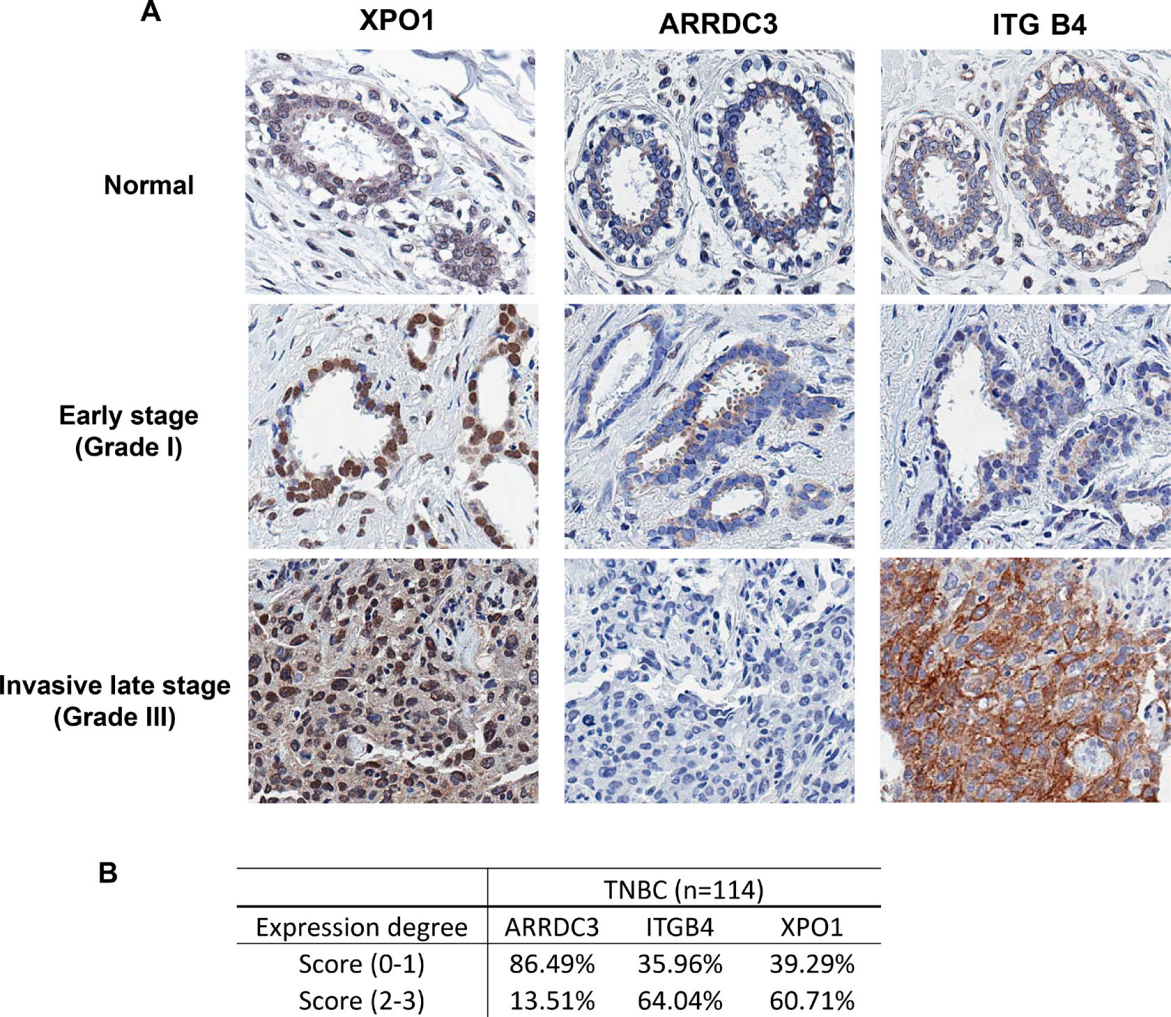
ARRDC3 expression is low in many TNBC tumors and inversely correlates with the levels of XPO1 and integrin β4 (**A**) Representative images of XPO1, ARRDC3 and ITG β4 expression in normal ductal epithelium (top panel), Grade I TNBC epithelium (middle panel), and Grade III TNBC epithelium (bottom panel), respectively. (**B**) The staining intensity of each tissue was scored (0–3) and categorized into either low (0–1) or high expression group (2–3) of XPO1, ARRDC3 and ITG β4.

To identify the sub-population of TNBC patients who may potentially benefit from treatment with selinexor, we assessed the levels of ARRDC3, XPO1 and ITG β4 from normal biopsy tissue as well as in 114 biopsies of TNBC patient tumor samples (Figure 4). Breast cancer tissue arrays were stained with anti-ARRDC3, anti-XPO1, and anti-ITG β4 antibodies for immunohistochemistry analysis. The representative staining of these 3 proteins in normal, early and late stage TNBC tumor tissues showed that ARRDC3 expression is either very low or undetectable in invasive stage relative to higher levels of expression in early stage or normal tissue, and inversely correlated with the levels of both XPO1 and ITG β4 (Figure 4A). Treatment of KPT-185 in MDA-MB-468 cells decreased the levels of ITG β4 in a dose and time course dependent manner, which further confirms the inverse correlation between ARRDC3 and ITG β4 levels (Supplementary Figure 3). The staining intensity of each tissue was scored (0–3) and categorized into either low (0–1) or high expression groups (2–3) (Figure 4B). We found that the majority (86.5%) of 114 TNBC patient tumors presented low ARRDC3 expression (Figure 4B). Therefore we predict that a significant portion of TNBC patients with low ARRDC3/high XPO-1/high ITG β4 sub-group are likely to respond to selinexor and therefore benefit from selinexor treatment.

### Selinexor effectively inhibited TNBC cell-derived xenograft tumors in mice while restoring ARRDC3 expression

**Figure 5:**
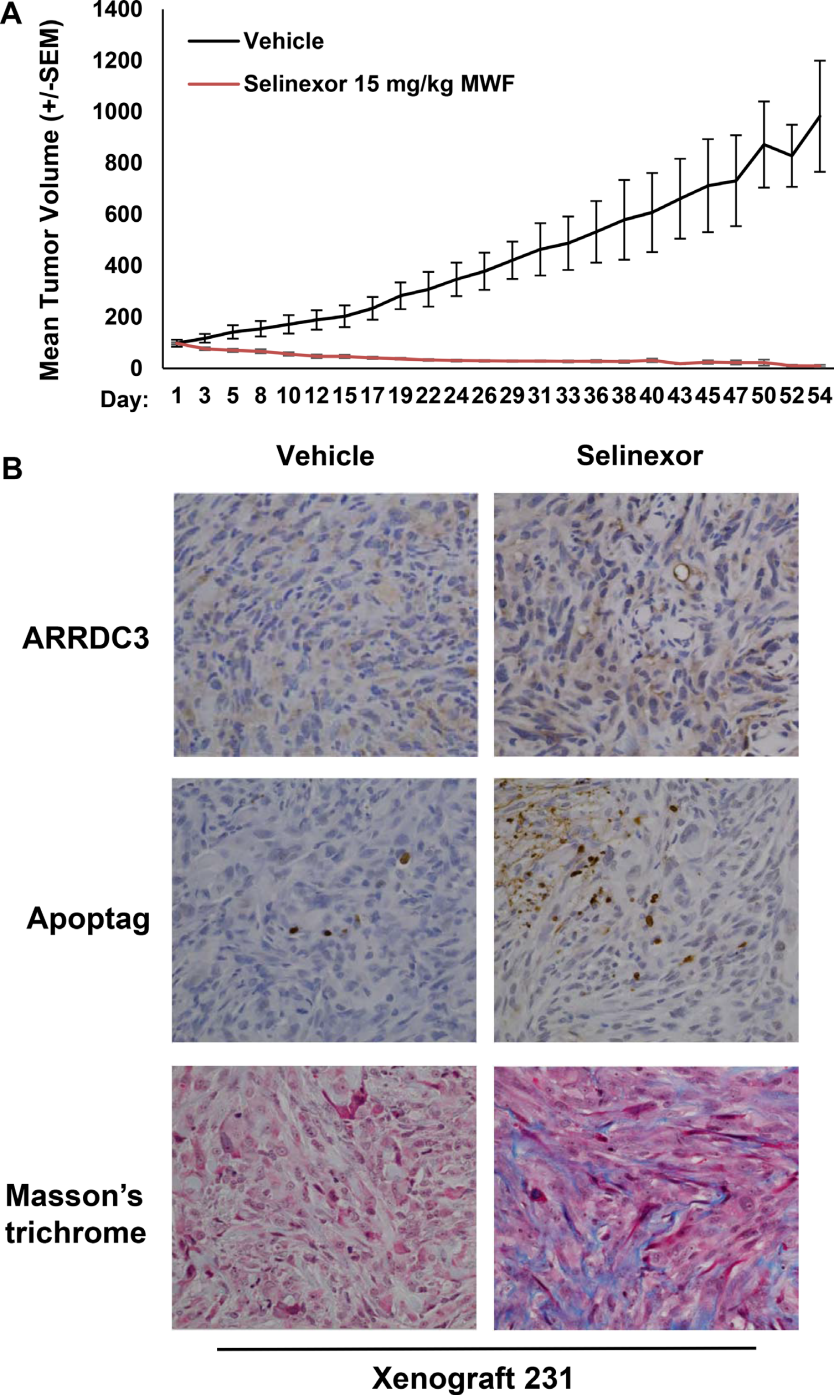
Selinexor effectively inhibited TNBC cell-derived xenograft tumors in mice while restoring ARRDC3 expression (**A**) Mice bearing MDA-MB-231 cell derived tumors were treated with vehicle or selinexor (15 mg/kg; PO, QOD: Monday, Wednesday and Friday). Tumor size (mm^3^) was measured at the indicated days for 54 days. Error bar represents SEM (*P* = 0.0002) (**B**) At the end of treatment, tumor tissues were excised. ARRDC3 and apoptosis were analyzed by immunohistochemically and tumor stroma by Mason's Trichrome.

In order to test the effects of selinexor *in vivo*, MDA-MB-231 tumor bearing mice were treated with 15 mg/kg of selinexor on a M,W,F schedule. Tumor growth and body weight were monitored for 54 days. Selinexor treatment resulted in 90% tumor growth inhibition over the course of treatment (Figure 5A). No overt evidence of toxicity was observed during the treatment except for minimal body weight loss. Immunohistochemical assessment of tumor sections collected 1 week post-selinexor treatment revealed increased numbers of apoptotic cells and increased fibrosis (Figure 5B) as evidenced by Apoptag (staining of apoptotic cells) and Masson's trichrome staining (staining of connective tissue). Consistent with *in vitro* findings, the restoration of ARRDC3 expression was seen in tumor specimens treated with selinexor. Overall, the xenograft studies confirm the efficacy of selinexor in a TNBC model and provide the basis for a biomarker (ARRDC3) based clinical trial of selinexor for the treatment of TNBC.

## DISCUSSION

TNBC is associated with early disease relapse and distal metastasis that leads to an overall poor prognosis [1–3]. As chemotherapy is the only systemic therapeutic option for a subset of chemotherapy sensitive TNBC patients, it is urgent to understand the subtype specific molecular nature that confers to chemo-resistance or sensitivity among TNBC patients, and apply these mechanisms for TNBC specific drug development. Our current studies highlighted the importance of ARRDC3 in mediating therapeutic effects of SINE compounds in a TNBC model. These studies support the novel inhibitory mechanisms of action of SINE compounds provide further evidence that SINE compounds could emerge as a novel therapeutic option for multiple cancers, including breast cancer [31–37].

Baseline expression of ARRDC3 may serve as a predictive marker to identify potential sensitive tumors and patients who may benefit from treatment with selinexor, a SINE compound, which currently in clinical trials [36, 37] (clinicaltrials.gov). Importantly, the sensitivity of breast cancer cells to SINE compounds may depend not only on ARRDC3 protein levels, but also on the availability of ARRDC3 downstream substrates and their phosphorylation status as ARRDC3 only interacts with phosphorylated form of their substrates [17]. MDA-MB-231 cells, a SINE compound-sensitive cell line, indeed overexpress the signaling active and phosphorylated form of ITG β4 [17], suggesting the potential connection of ARRDC3/ phospho-ITG β4 levels in the sensitivity of SINE compounds. Our TMA analysis further supports this hypothesis by showing the inverse correlation between ARRDC3 and ITG β4 levels among TNBC tumor tissues. While more studies are needed to identify the breast cancer subtypes that are sensitive to SINE compounds, our result suggest that TNBCs that express lower ARRDC3 levels are more sensitive and that SINE compounds can emerge as a therapeutic option for TNBC as a single agent or as part of a combination therapy.

The mechanism by which SINE compounds restore the expression of ARRDC3 remains to be determined. As multiple tumor suppressors and nuclear transcription regulators are accumulated in the nucleus as a function of SINE compounds, it will be challenging to pinpoint out the single mechanism by which ARRDC3 transcription is restored in TNBC cells. It is also possible that multiple mechanisms may synergize to enhance ARRDC3 transcription. The leading candidate to mediate ARRDC3 expression by SINE compounds is Sirt2 that plays a major role in epigenetic silencing of ARRDC3 in TNBC cells [14]. Earlier studies suggest that Sirt2 is indeed an XPO1 cargo and therefore selinexor treatment is expected to induce its nuclear localization and activate its function [38]. Our preliminary studies did not provide evidence that SINE compounds change the levels or nuclear localization of Sirt2. Post-translational modifications of Sirt2 by SINE compounds can be a potential mechanism. Therefore additional studies of Sirt2 will be at the focus of our future experiments.

In conclusion, the current pre-clinical studies demonstrated the efficacy of selinexor in TNBC model *in vitro* and *in vivo*, and will provide the basis for potential clinical trial of SINE compounds in TNBC patients with low ARRDC3 expression.

## MATERIALS AND METHODS

### Cell lines and reagents

MDA-MB-231 and MDA-MB-468 breast adenocarcinoma cells were purchased from the American Type Culture Collection and cultured in DMEM 1 g/L glucose, L-glutamine and sodium pyruvate formulation, supplemented with 10% FBS and 1% penicillin/streptomycin. Cells were cultured in humidified incubators at 37°C in 5% CO_2_. To generate ARRDC3 knockdown stable cell lines, cells were infected with lenti-virus expressing shRNA targeted against either GFP (as control) or ARRDC3 (Sigma, St. Louis, MO). The infected cells were then selected by puromycin (20 μg/ml). SINE compounds (KPT-185 and KPT-330; selinexor) were provided by Karyopharm therapeutics, Inc (Natick, MA). 10 mM stocks of the inhibitors were diluted in medium to the indicated concentrations prior to the treatment of cells.

### Western blot analysis

Cells were lysed in cold RIPA-EDTA buffer [50 mM Tris, pH 7.4; 150 mM NaCl; 1% NP-40; 0.5% sodium deoxycholate; 0.1% SDS; and 5 mM EDTA] containing 1 mM phenylmethylsulfonyl fluoride, 1 mM Na_3_VO_4_, and protease inhibitor (Thermo Scientific, Rockford, IL). The protein concentrations were determined using the BCA protein assay kit (Thermo Scientific). The samples were separated on 4% to 20% gradient SDS PAGE. The separated proteins were transferred to PVDF transfer packs using the Trans-Blot Turbo transfer system (Bio-Rad, Hercules, CA). The blots were incubated with primary antibodies in TBS-T or TBS-T with 5% w/v nonfat dry milk overnight at 4°C, and then with appropriate secondary antibodies conjugated to IgG-horseradish peroxidase. CRM-1 (H-300) and beta-actin (C-11) antibodies were purchased from Santa Cruz Biotechnology. ARRDC3 antibody was obtained from Abcam. Proteins were detected using a Clarity Western ECL substrate (Bio-Rad). All bands were imaged with ChemiDoc Touch Imaging System (Bio-Rad).

### Quantitative RT-PCR

The real time PCR Taqman gene assay for ARRDC3 (ID:Hs00385845_m1; ccacctctttattcagagattgatc) and GPADH (ID:Hs99999905_m1; gggcgcctggtcaccagggctgctt) were purchased from Life Technologies. MDA-MB-231 and MDA-MB-468 cells were treated with various concentrations of KPT-185 and KPT-330 for either 4 or 24 hours. RNA was extracted from cells using RNeasy Kit (#74106, Qiagen) and reverse transcribed to cDNA using High Capacity cDNA Reverse Transcription Kit (#4368813, Applied Biosystems) following manufacturer's protocol. mRNAs for the indicated genes were quantified using ViiA7 Real-Time PCR system and analyzed by the V1.2 software (Life Technologies).

### Cell motility assay

Cell motility assays were performed by a transwell cell culture chamber of 8 μm pore size (Costar-Falcon) according to the standard procedure. Transwell inserts were coated with collagen I (15 μg/ml) overnight at 4°C. After washing the inserts with PBS next day, cells were added to the upper chamber of each well. LPA (Lysophosphatidic acid; 100 ng/mL) was added to the lower chambers as a chemoattractant. The chambers were incubated for 2 h at 37°C with 10% CO_2_. The cells that did not migrate through the pores were mechanically removed by cotton swab. The migrated cells on the lower surface of the membrane were fixed and stained with 0.2% crystal violet and counted. Assays were performed in triplicate and repeated three times.

### Cell proliferation assay

Cells (2.5 × 10^3^) were seeded in 96-well plates with 100 μL media in triplicate and allowed to adhere overnight. Drug containing media was added to each well at the concentrations indicated. After the treatment for 48 or 72 h, viability was evaluated using the Kit-8 (CCK-8, Enzo Life Sciences, Switzerland) according to the manufacturer's instructions. Absorption at 450 nm was determined using a Perkin Elmer EnVision^®^ Multilabel Reader.

### Soft agar growth assay

ARRDC3 or control shRNA expressing MDA-MB-231 cells were suspended in the top layer of DMEM (1 mL) containing 0.35% low-melt agarose (ISC Bioexpress) with or without SINE compound, and then the top layer was overlaid on DMEM (2 mL) containing 0.75% agar in six-well plates. The cells were fed twice per week with 0.5 mL DMEM in the presence of SINE compound. After 3 weeks, the total number of colonies was quantified by counting 50 fields per well by using bright-field optics. The average number of colonies was obtained from counting triplicate wells. The images of colonies were acquired by a microscope and digital camera (Nikon).

### Wound-healing assay

MDA-MB-231 cells were loaded into each well of Culture-inserts (Ibidi, Bonn, Germany) at 2 × 10^5^ cells/well and allowed to adhere overnight. The insert was removed and attached cells were washed twice with 1x PBS. SINE compound was then added to the plate. The progression of wound closure and the cells migrated into empty spaces of wound were determined under an inverted phase-contrast microscope with a distal camera (Nikon). Images were captured at indicated time points. Wound-healing assays were carried out in triplicates.

### Xenograft model

Twenty (20) nu/nu mice were inoculated subcutaneously with 2 × 10^7^ MDA-MB-231 cells. Treatment was initiated when the tumors reached a mean volume of 80 mm^3^ (standard deviation ± 28.7 mm^3^ range 50–157 mm^3^). Mice were allocated to 2 groups of ten (10) mice such that mean tumor volume in each group was within the range of 69 to 98 mm^3^. Mice were treated with vehicle or selinexor at 15 mg/kg on a Monday, Wednesday and Friday (MWF) schedule. Body weight and condition of each animal were recorded daily, and tumors were measured on MWF.

### Immunohistochemistry

Formalin fixed paraffin embedded (FFPE) tissue blocks were sectioned at 4 microns, and deparaffinized through three changes of xylenes and a decreasing series of ethanol. Antigen retrieval was performed in a steam cooker for 15 minutes in Declere (Cell Marque) working solution. Endogenous hydrogenase was blocked by incubation in 3% Hydrogen Peroxide for five minutes. Slides were incubated in Casein-based protein block (Biogenex) for 20 minutes before incubation with ARRDC3 antibody (Abcam) at room temperature for 30 minutes. Slides were then rinsed with buffer and incubated with Amplifier from Hi-Def Polymer Detection Kit (Cell Marque) for 10 minutes at room temperature. Afterwards slides were rinsed with buffer and incubated in DAB chromogen for six minutes at room temperature for color development. The slides were counterstained with Hematoxylin I (Richard Allan Scientific), rinsed in water, and dehydrated through a series of increasing ethanol and three changes of xylenes. Slides were then coverslipped. Digital images of slides were generated via Aperio AT scanner at 20×. Immunohistochemistry assays were performed on a Biogenex I6000 automated stainer. The apoptag apoptosis assay (Millipore, Cat No. 7100) and Masson's Trichrome (Polyscientific) staining were performed manually per the manufacturer's instructions.

### Tissue microarray (TMA) analysis

Commercial breast cancer TMA slides BRC964 and BRC1021 from Pantomics were stained by IHC with XPO1 (Santa Cruz, sc-5595), AARDC3 (Abcam, ab64817) and ITG β4 (Santa Cruz, sc-9090) antibodies. All breast cancer cases were reviewed and scored by a pathologist.

## SUPPLEMENTARY FIGURES


