# Supplementary material for: Selective Inhibitors of Nuclear Export (SINE) compounds block proliferation and migration of triple negative breast cancer cells by restoring expression of ARRDC3
Source: Oncotarget. 2017 May 18;8(32):52935–47. doi: 10.18632/oncotarget.17987 (PMC5581083; doi:10.18632/oncotarget.17987)
Supplement: Supplementary file 1 [file oncotarget-08-52935-s001.pdf]

Selective Inhibitors of Nuclear Export (SINE) compounds block proliferation and migration of triple negative breast cancer cells by restoring expression of ARRDC3

Supplementary Materials

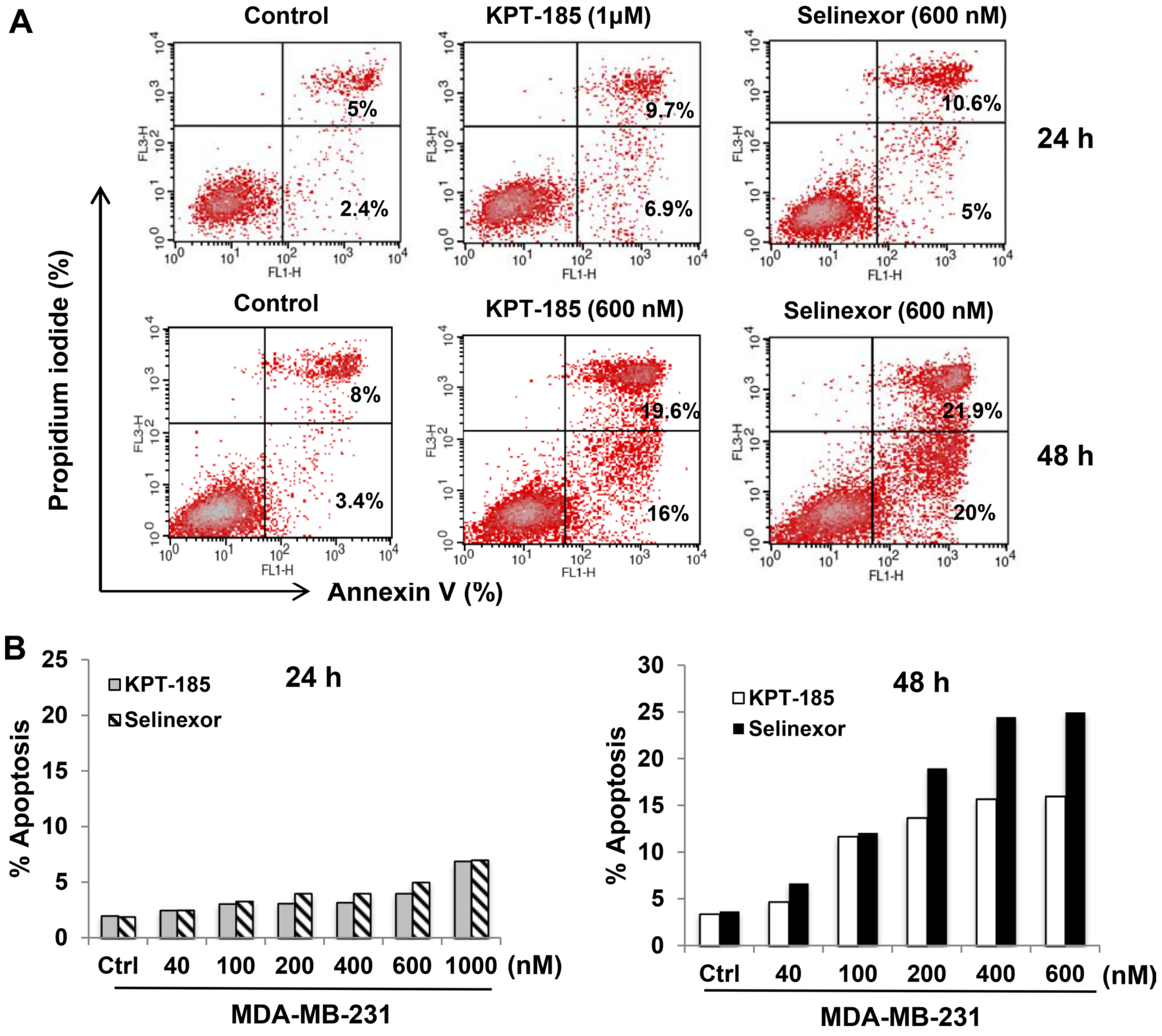

Supplementary Figure 1: No initiation of cell death is observed by SINE compounds at 24 hour treatment or under doses lower than 400 nM. (**A**) Apoptosis measured by flow cytometry. MDA-MB-231cells were treated with KPT-185 and Selinexor at the indicated concentrations for 24 h and 48 h and stained with Annexin V and propidium iodide. Early apoptotic cell populations are shown in the lower right quadrant and late apoptotic cell populations are shown in the upper right quadrant. (**B**) Representative graph shows the average percentage of early apoptotic cells in each case.

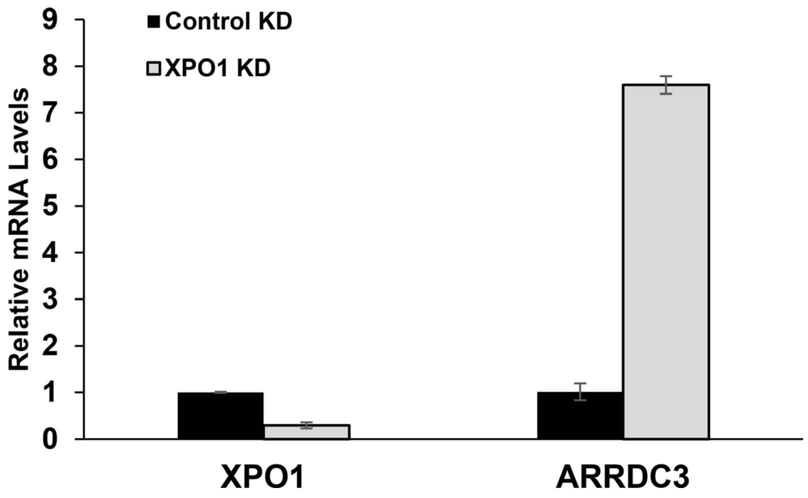

Supplementary Figure 2: Knocking down of XPO1 results with direct upregulation of ARRDC3 expression. siRNA was used to knock down the expression of XPO1 and expression levels of both XPO1 and ARRDC3 mRNA were quantified by Real-time PCR. U-2 OS cells were transfected with XPO1 siRNA (40 nM) and 48 hours later total RNA was purified and expression levels of XPO1 and ARRDC3 mRNA were quantified by Real-time PCR. The results show that 80% silencing of XPO1 mRNA levels resulted with 7.5-fold induction of ARRDC3.

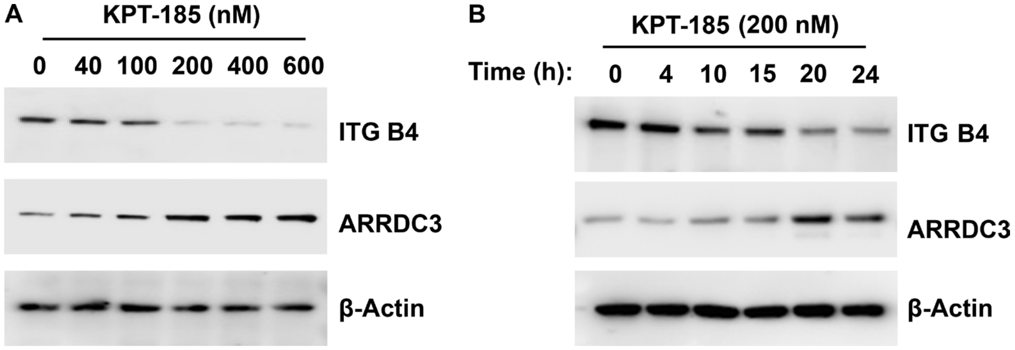

Supplementary Figure 3: KPT-185 decreases the levels of integrin β4 (ITG β4) in a dose and time course dependent manner. (**A**) MDA-MB-468 cells were treated with or without various concentrations of KPT-185 for 24 h. (**B**) MDA-MB-468 cells were treated with KPT-185 (200 nM) at the indicated times. Protein levels of ITG β4 , ARRDC3 and β-actin were determined by western blot analysis.
